# Supplementary material for: Isothiocyanate-Functionalized Mesoporous Silica Nanoparticles as Building Blocks for the Design of Nanovehicles with Optimized Drug Release Profile
Source: Nanomaterials (Basel). 2019 Aug 29;9(9):1219. doi: 10.3390/nano9091219 (PMC6780844; doi:10.3390/nano9091219)
Supplement: Supplementary file 1 [file nanomaterials-09-01219-s001.pdf]

Isothiocyanate-Functionalized Mesoporous Silica Nanoparticles as Building Blocks for the Design of Nanovehicles with Optimized Drug Release Profile

Gabriel Martínez-Edo [1], Maria C. Llinàs [1], Salvador Borrós [1,2] and David Sánchez-García [1,*]

[1] Grup d'Enginyeria de Materials (GEMAT), Institut Químic de Sarrià, Universitat Ramon Llull, Via Augusta, 390, 08017 Barcelona, Spain; gabrielmartineze@iqs.url.edu (G.M.-E.); mariallinasr@iqs.url.edu (M.L.); salvador.borros@iqs.url.edu (S.B.)

[2] Centro de Investigación Biomédica en Red en Bioingeniería, Biomateriales y Nanomedicina (CIBER-BBN), 50018 Zaragoza, Spain

Table of Contents

I. Characterization of MSN-(NH_2) and MSN-(NCS) S2

II. Functionalization test of MSN-(NCS) and MSN-(N_3) S4

III. Characterization of regioselective bifunctionalized MSN-$(NH_2)_i(NCS)_o$.......... S6

I. Characterization of MSN-(NH_2) and MSN-(NCS)

Table S1. Dynamic light scattering (DLS) size and Z-potential values of MSN-(NH_2) and MSN-(NCS) of 50 and 100 nm.

Size / nm	TEM	DLS	PDI	Z-pot / mV
MSN-(NH_2)	50	129	0.19	-1.7
	100	142	0.07	-12
MSN-(NCS)	50	152	0.09	-1.4
	100	190	0.19	-11

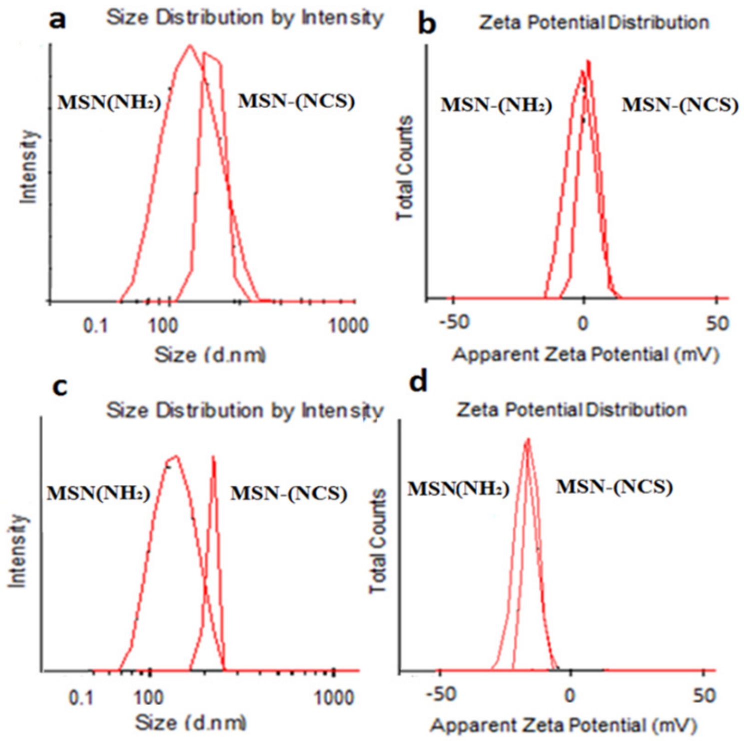

Figure S1. Dynamic light scattering (DLS) size and Zeta-potential values of MSN-(NH_2) and MSN-(NCS) of 50 nm (a, b) and 100 nm (c, d).

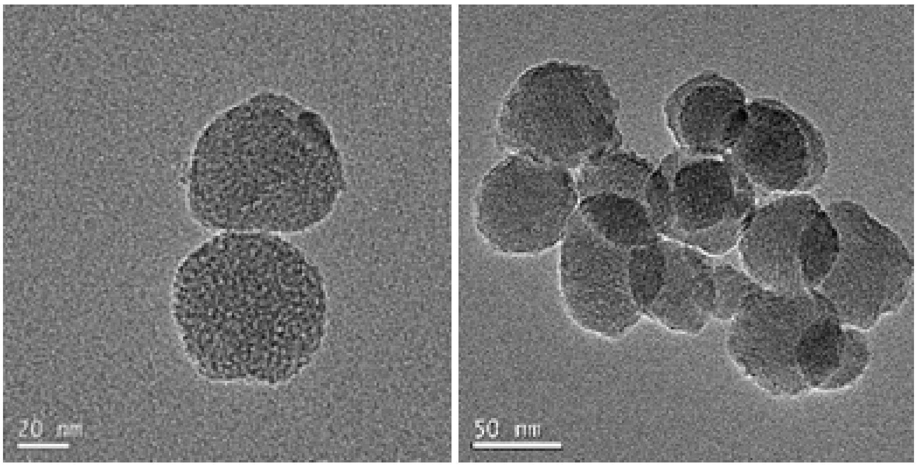

Figure S2. Representative TEM images of 50 nm MSN-(NCS).

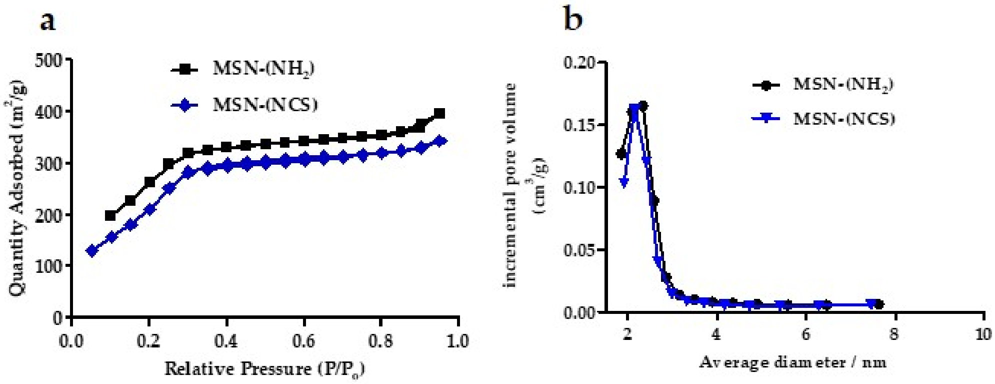

Figure S3. N_2 adsorption-desorption and BJH pore size distribution plots of of MSN-(NH_2) and MSN-(NCS) of 50 nm (a, b).

Table S2. N_2 adsorption-desorption and BJH pore size distribution values of MSN-(NH_2) and MSN-(NCS) of 50 nm.

	**MSN-(NH_2)**	**MSN-(NCS)**
**BET surface area (m^2/g)**	1120.90	849.20
**BJH pore volume (cm^3/g)**	0.72	0.53
**Pore size (nm)**	2.30	2.20

II. Functionalization test of MSN-(NCS) and MSN-(N_3)

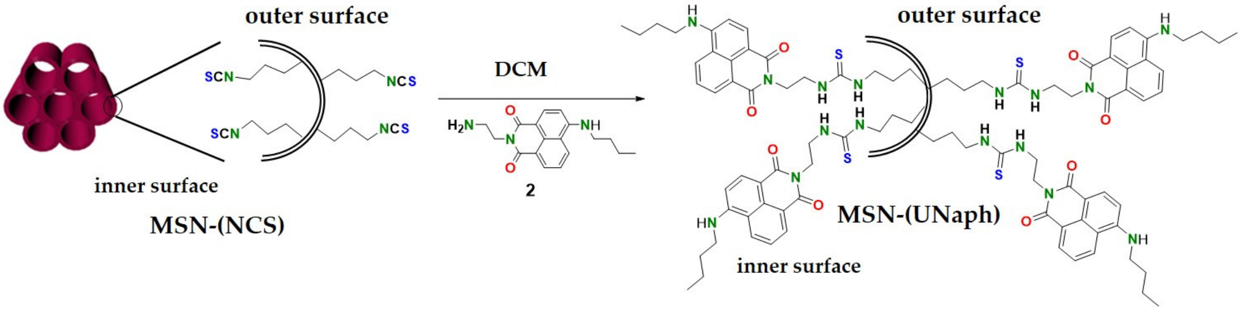

Figure S4. MSN-NCS functionalization with 4-(*n*-butylamino)-*N*-(2-aminoethyl)-1,8-naphthalimide.

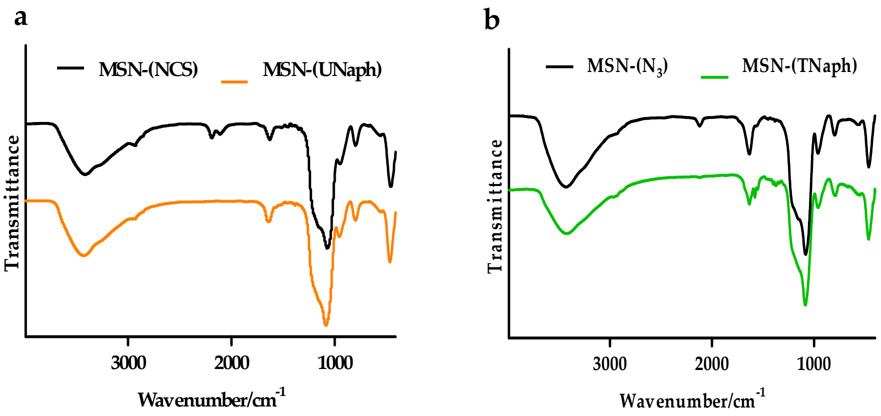

Figure S5. FTIR spectra of MSN-(NCS) (a) and MSN-(N_3) (b) reacted with 4-(*n*-butylamino)-*N*-(2-aminoethyl)-1,8-naphthalimide.

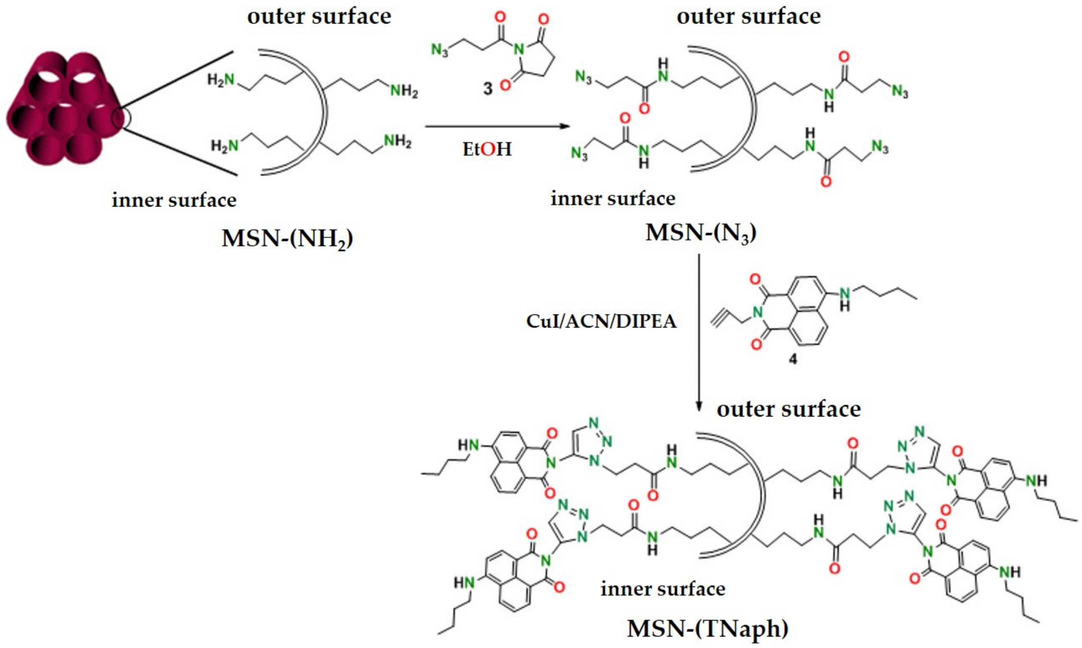

Figure S6. MSN-(N_3) functionalization with 4-(*n*-Butylamino)-*N*-(2-propargyl)-1,8-naphthalimide.

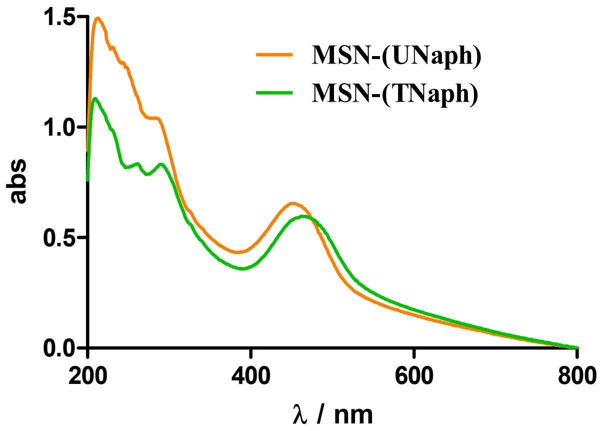

Figure S7. Absorption spectra of MSN-(UNaph) and MSN-(TNaph).

III. Characterization of regioselective bifunctionalized MSN-$(NH_2)_i(NCS)_o$

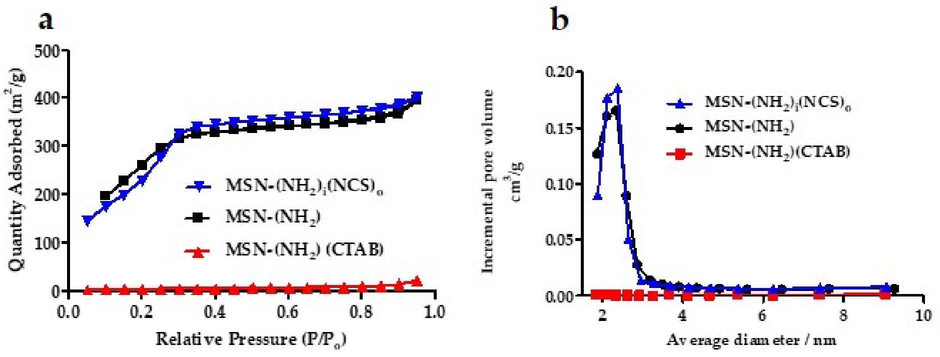

Figure S8. N_2 adsorption-desorption surface area (a) and BJH pore size distribution plots (b) of MSN-(NH_2) (CTAB), MSN-(NH_2) and MSN-$(NH_2)_i(NCS)_o$ of 50 nm.

Table S3. N_2 adsorption-desorption and BJH pore size distribution values of MSN-(NH_2) (CTAB), MSN-(NH_2) and MSN-$(NH_2)_i(NCS)_o$ of 50 nm.

	**MSN-NH_2(CTAB)**	**MSN-(NH_2)**	**MSN-$(NH_2)_i(NCS)_o$**
**BET surface area (m^2/g)**	78.60	599.80	554.50
**BJH pore volume (cm^3/g)**	0.25	0.55	0.45
**Pore size (nm)**	--	2.60	2.60

Table S4. Dynamic light scattering (DLS) size and Z-potential values of MSN-(NH_2) and MSN-$(NH_2)_i(NCS)_o$ of 50 nm and 100 nm.

**Size / nm**	**TEM**	**DLS**	**PDI**	**Z-pot / mV**
**MSN-(NH_2)**	50	129	0.19	-1.7
	100	142	0.07	-12
**MSN-$(NH_2)_i(NCS)_o$**	50	141	0.29	-1.7
	100	173	0.04	-13

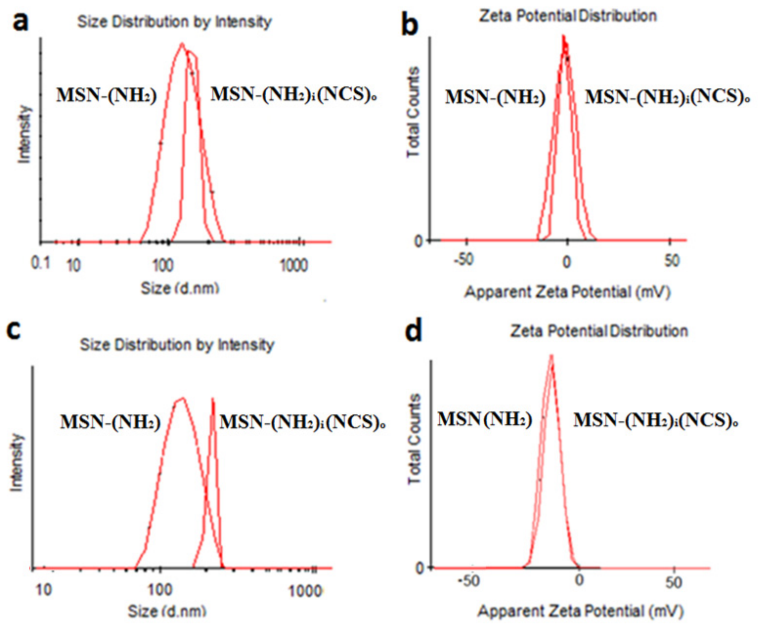

Figure S9. Dynamic light scattering (DLS) size and Z-potential measures of MSN-(NH_2) and MSN-$(NH_2)_i(NCS)_o$ of 50 nm (a, b) and 100 nm (c, d).

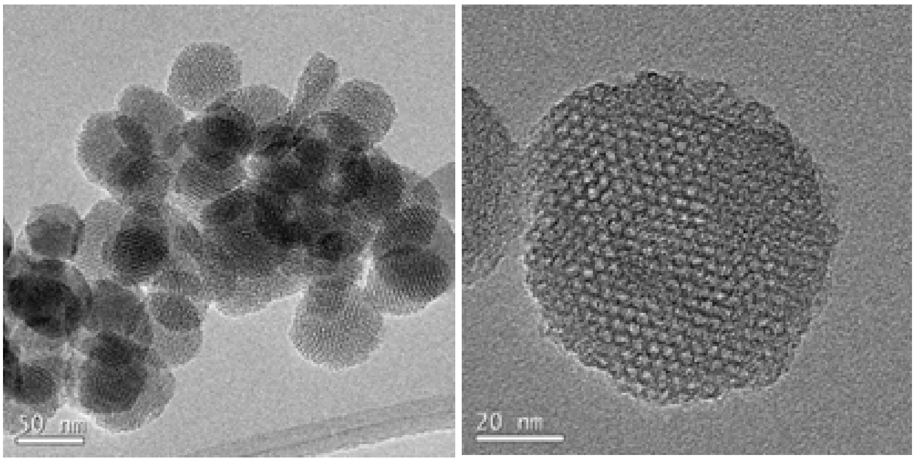

Figure S10. TEM images of 50 nm MSN-$(NH_2)_i(NCS)_o$.

$$\%\ Ataluren\ relaesed = \frac{mg\ Ataluren\ released}{mg\ Ataluren\ charged} \cdot 100$$

Equation S1. % Release Ataluren.
